# Fabrication and Property Regulation of Small-Size Polyamine Microcapsules via Integrating Microfluidic T-Junction and Interfacial Polymerization

**DOI:** 10.3390/ma14071800

**Published:** 2021-04-05

**Authors:** Shaochuan Lai, Yongjun He, Daoying Xiong, Yao Wang, Kaibin Xiao, Zhibin Yan, He Zhang

**Affiliations:** 1 South China Company of National Petroleum and Natural Gas Pipe Network Group, Guangzhou 510620, China; laisc@pipechina.com.cn (S.L.); heyj@pipechina.com.cn (Y.H.); xiongdy@pipechina.com.cn (D.X.); wangy@pipechina.com.cn (Y.W.); 2 National Engineering Research Center of Novel Equipment for Polymer Processing, Key Laboratory of Polymer Processing Engineering (SCUT), Ministry of Education, South China University of Technology, Guangzhou 510641, China; 201920101613@mail.scut.edu.cn; 3 Guangdong Provincial Key Laboratory of Technique and Equipment for Macromolecular Advanced Manufacturing, South China University of Technology, Guangzhou 510641, China; 4 Guangdong Provincial Key Laboratory of Optical Information Materials and Technology & Institute of Electronic Paper Displays, South China Academy of Advanced Optoelectronics, South China Normal University, Guangzhou 510006, China; zhibin.yan@m.scnu.edu.cn

**Keywords:** polyamine, microcapsule, T-junction, microfluidic, interfacial polymerization

## Abstract

The self-healing system based on microencapsulated epoxy-amine chemistry is currently the self-healing system with the most practical application potential. It can be widely used in many epoxy-based materials with a size restriction for the microcapsules, such as fiber-reinforced composites, anti-corrosion coatings, etc. Although epoxy microcapsules of different sizes can be fabricated using different techniques, the preparation of polyamine microcapsules with suitable sizes and good performance is the prerequisite for further developing this self-healing system. In this investigation, based on the novel microencapsulation technique via integrating microfluidic T-junction and interfacial polymerization, the feasibility of preparing small-size polyamine microcapsules and the process regulation to optimize the properties of the small-size microcapsules were studied. We show that polyamine microcapsules with sizes smaller than 100 μm can be obtained through the T-junction selection and the feeding rate control of the polyamine. To regulate the small-size microcapsules’ quality, the effects of the concentration of the shell-forming monomer and the solvent with different polarity in the reaction solution and the reaction condition were studied. It shows that dry, free-flowing small-size microcapsules can still be obtained when the shell-forming monomer concentration is higher and the solvent’s polarity is lower, compared with the preparation of larger polyamine microcapsules. Although the change of reaction conditions (reaction temperature and duration) has a certain effect on the microcapsules’ effective core content, it is relatively small. The results of this investigation further promote the potential application of the self-healing systems based on microencapsulated epoxy-amine chemistry in materials with a size restriction for the microcapsules.

## 1. Introduction

Polymeric materials suffer from local damages and microcracks inevitably during their life cycle, which finally cause macrocracks and even fractures to affect their normal use, shorten their service life, and increase safety risk, posing a huge threat to the safety of people’s lives and properties. To early prevents the small injuries from evolving into big damages, various techniques have been developed, [1,2,3,4,5,6], among which the self-healing technique is the most attractive and prospective one [5,7,8,9]. The self-healing of materials is the ability of materials to fully autonomously repair damages and restore lost or degraded properties or performance using resources inherently available to the system without any external interventions. Since the self-healing technique has the advantages of extending the service life of materials, improving product safety, and reducing material maintenance costs, it has a wide range of application prospects in the fields of aircraft, automobiles, construction, military equipment, electronic products, etc. To date, with the continuous development and maturity of self-healing techniques, one trend in this field is how to practically apply this technique and promote its practicality and commercialization. [5,10] Attributed to the ease of preparation of healant carriers, a wide selection of self-healing chemistry, and easy integration with commercial polymer resins, the self-healing systems based on microcapsules are thought to be the most practical self-healing systems among all the developed extrinsic self-healing systems [11,12,13].

The self-healing systems with the potential to be practical applied should not only have the characteristics of low toxicity, low cost, healing fast and efficiently, matrix compatibility, long-term stability, etc., but also have less impact on the performance of the matrix and ease of being integrated with the matrix for specific applications. For the microcapsule-based self-healing systems, the use of smaller microcapsules can reduce the influence of the added microcapsules on the mechanical properties of the matrix, compared with larger microcapsules [14,15,16,17]. Taking the self-healing fiber-reinforced composite that is widely used to verify healability, for example, the microcapsule not only needs to fulfill the self-healing function but also needs to be compatible with the manufacturing process of the composite reinforced by fibers [18,19]. Considering the limited space among different fibers and different fiber bundles, and between fiber layers, the microcapsule size is a key factor. In addition, when preparing self-healing materials with limited shape and size, such as self-healing anti-corrosion coatings, the microcapsules’ particle size is also a critical factor since coatings normally are relatively thin [20]. However, to obtain better healing performance in these self-healing materials, most of the investigations used large-size microcapsules with high core content for technical verification. Although there are also studies using small-size microcapsules or even nanocapsules, [21,22,23] they are not deliberately carried out for practical purposes.

The self-healing epoxy system based on microencapsulated two-part epoxy-amine chemistry has been considered as a practical self-healing system, owing to the characteristics of low toxicity, cost-effectiveness, matrix compatibility, healing fast with high performance, and environmental stability [10,24,25,26,27,28,29,30,31,32]. Although the microencapsulation of epoxy monomers can be achieved by various techniques, [25,26,27,31] the microencapsulation of polyamine hardeners is extremely challenging attributed to their high reactivity, amphiphilicity, and good solubility in water and most organic solvents [24,25,27,31,33,34,35]. Thus, it becomes the bottleneck for the further development of this self-healing system. In 2018, Zhang et al. [10] made a breakthrough for the microencapsulation of polyamines using a novel technique via integrating microfluidic T-junction and interfacial polymerization and fabricated a high-performance self-healing epoxy with great practicality. However, to achieve high healing performance, they mainly used polyamine microcapsules with a size of about 200 μm. To better verify the system’s practicality, it is of great necessity to fabricate smaller polyamine microcapsules to further the study.

Considering the difficulty in the fabrication of polyamine microcapsules, it will be much more challenging to prepare small-size polyamine microcapsules on demand. Based on the microencapsulation technique via integrating microfluidic T-junction and interfacial polymerization, this investigation attempts to verify the feasibility in preparing polyamine microcapsules with a smaller size and further optimize the quality of the small-size polyamine microcapsules.

## 2. Experiment

### 2.1. Materials

Tetraethylenepentamine (TEPA) and 1,4-diazabicyclo [2.2.2]octane (DABCO) were purchased from Shanghai Macklin Biochemical Co. Ltd., Shanghai, China. The used solvent, including *n*-hexadecane (C16), decalin (C10), and cyclohexane (C6), were purchased from Alfa-Aesar, Haverhill, MA, USA. The non-ionic surfactant, Arlacel P135, was purchased from Croda International Plc., Snaith, UK. The adopted tri-functional polyether amine, JEFFAMINE T403, was provided by Huntsman International LLC., Salt Lake City, UT, USA. The adopted shell-forming monomer, 4,4’-methylenebis(cyclohexyl isocyanate (HMDI), was purchased from Yantai Wanhua Polyurethane Co., Ltd., Yantai, China. All the chemicals were used as received without any other treatments.

**Figure 1:**
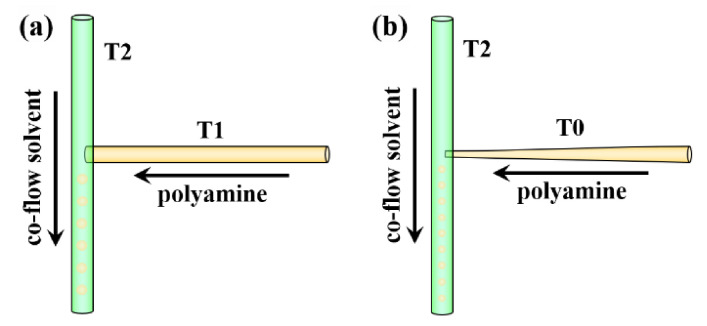
Configuration of the adopted T-junction units. (**a**) T-junction T1/T2, and (**b**) T-junction T0/T2.

The Teflon tubing to manufacture the T-junction units, including T1 with an inner diameter (ID) of 100 μm and outer diameter (OD) of 400 μm and T2 with ID of 300 μm and OD of 760 μm, was purchased from Microbore. The T-junction T1/T2 was fabricated by inserting T1 into T2, as shown in Figure 1a. To fabricate the T-junction unit that can generate smaller polyamine droplets and thus fabricate smaller polyamine microcapsules, tubing T1 was stretched to further reduce the diameter (labeled as T0) and inserted into T2 to manufacture T-junction T0/T2 (Figure 1b). In this investigation, the I.D. of T0 was about 50 μm.

### 2.2. Fabrication of Small Polyamine Microcapsules

The fabrication of small-size polyamine microcapsules was carried out using the technique via integrating microfluidic T-junction and interfacial polymerization, which was successfully demonstrated in our group previously [10,36]. Briefly, the co-flow solvent, C16 with 1.0 wt% surfactant Arlacel P135, and the polyamine mixture, 25 wt% TEPA and 75 wt% JEFFAMINE T403 (25TEPA75T403), were separately injected through the T-junction unit at feeding rates of 15.0 mL/h and ***V*** mL/h, respectively using two syringe pumps (Lead Fluid, TYD02) to generate polyamine droplets, as shown in Figure 1. Once the polyamine droplets, together with the co-flow solvent, flowed into the reaction solution consisting of 50.0 mL nonpolar solvent, ***M*** g HMDI, 1.0 wt% Arlacel P135, and 0.5 wt% catalyst DABCO, they were microencapsulated by polyurea membrane formed through the rapid interfacial polymerization between polyamine molecules and HMDI to generate the preliminary polyamine microcapsules. In this shell-forming stage, the reaction solution was continuously stirred slightly at a speed of about 120 rpm using a three-blade propeller with a diameter of about 60 mm. After the addition of 12.0 mL co-flow solvent and the corresponding volume of the polyamine, the feeding process was terminated, and the preliminary polyamine microcapsules were separated and transferred into a new reaction solution consisting of 50.0 mL C6, 6.0 g HMDI, 1.0 wt% Arlacel P135, and 0.5 wt% DABCO. Under continuous agitation at 200 rpm using the above-mentioned three-blade propeller, the preliminary microcapsules thicken their shells to become the final polyamine microcapsules under certain reaction conditions (combinations of reaction temperatures and the corresponding durations). After this shell-growth stage, the polyamine microcapsules were rinsed using pure C6 for about 6–8 times and dried at room temperature (RT, ~22–25 °C) for about 5–10 min.

The adopted experimental parameters, including the adopted T-junction unit, the feeding rate for the polyamine (***V***, mL/h), the amount of HMDI (***M***, g) and the type of the nonpolar solvent (***S***) in the reaction solution, and the reaction conditions (combinations of reaction temperatures and the corresponding durations) to thicken the microcapsule shell, were tabulated in Table 1 for easy reference.

### 2.3. Characterization Methods

In this investigation, the morphology, size distribution, and shell structure of the synthesized polyamine microcapsules were characterized using a scanning electron microscope (SEM, FEI QUANTA FEG 250, New York, NY, USA). The size of the synthesized microcapsules was measured from SEM images using software, Image J, based on at least 30 individuals. The composition and thermal properties of the microcapsules were characterized using thermogravimetric analysis (TGA, Netzsch TG209-F3, Selb, Germany). During testing, about 10.0 mg samples were loaded in a platinum pan and heated up to 500 °C at a heating rate of 10 °C/min under a nitrogen atmosphere.

## 3. Results and Discussion

### 3.1. Size Regulation of Polyamine Microcapsules

**Figure 2:**
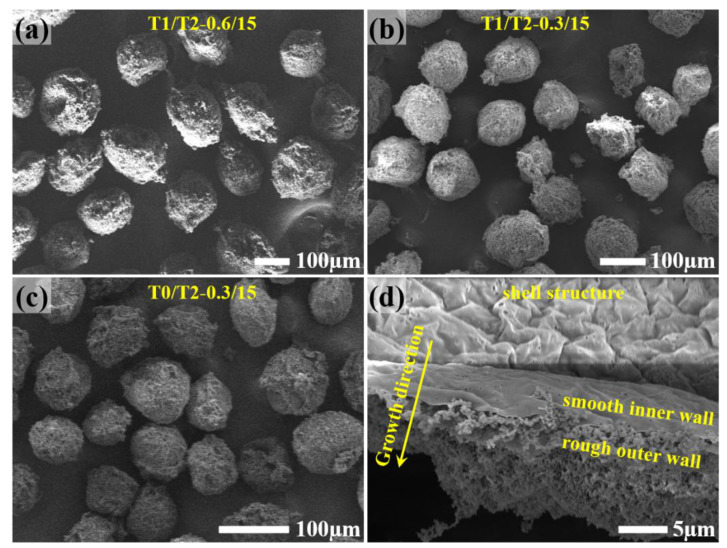
SEM images of the synthesized polyamine microcapsules using different T-junction units and different feeding rates for the polyamine. (**a**) Microcapsules synthesized using T1/T2 with a feeding rate of 0.6 mL/h for the polyamine; (**b**) microcapsules synthesized using T1/T2 with a feeding rate of 0.3 mL/h for the polyamine; (**c**) microcapsules synthesized using T0/T2 with a feeding rate of 0.3 mL/h for the polyamine; and (**d**) cross-section of the microcapsules synthesized using T1/T2 with a feeding rate of 0.3 mL/h for the polyamine.

**Figure 3:**
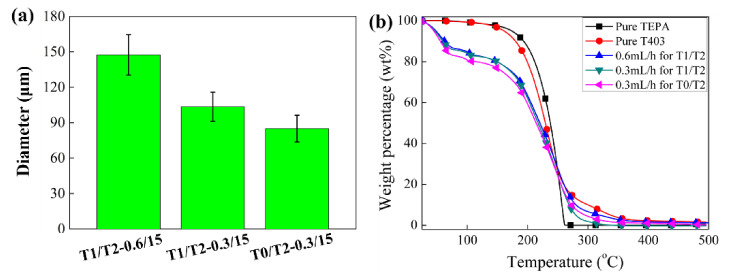
(**a**) Diameters of the synthesized polyamine microcapsules when different T-junctions and different feeding rates of the polyamine were adopted, and (**b**) TGA curves of pure tetraethylenepentamine (TEPA), pure T403, and the microcapsules with different sizes.

First, the feasibility of fabricating small-size polyamine microcapsules was studied by using T-junction T1/T2. It shows that dry, free-flowing microcapsules can be synthesized when the feeding rate of the co-flow solvent is 15.0 mL/h, and the feeding rate for the polyamine is 0.6 or 0.3 mL/h. Figure 2a,b shows the microcapsules at these two feeding rates of the polyamine, respectively. The surface of the microcapsules is relatively rough, and the shape is much more irregular in comparison with the big microcapsules (200–250 μm) containing the same polyamine synthesized using the same parameters except the feeding rate for the polyamine. According to the statistical data shown in Figure 3a, the diameters of the two microcapsules are 147 ± 17 μm and 103 ± 13 μm, respectively. Figure 2d shows the cross-section of the microcapsule shell when the feeding rate for the polyamine is 0.3 mL/h. It can be seen that the microcapsule only has a thin, smooth inner wall and a thick rough outer wall, with the absence of the porous middle layer. This is consistent with our previous results when pure TEPA was encapsulated [36]. With decreasing the droplet size for the polyamine, the Laplace pressure exerted on the droplet increases, suppressing the self-emulsification of the polyamine and the generation of the secondary polyamine micro-droplets around. Thus, the secondary microcapsules in the microcapsule shell decrease dramatically or even disappear accordingly, leading to the disappearance of the porous middle layer.

To further reduce the microcapsule size, in principle, we can still use the T-junction T1/T2 by further reducing the feeding rate of the polyamine. However, it also reduces the production efficiency of the microencapsulation technique to fabricate microcapsules. According to our previous studies, [36,37] the use of smaller tubing to feed polyamines can also generate smaller droplets and thus prepare small-size polyamine microcapsules. Therefore, in this investigation, the T-junction T0/T2, manufactured using Teflon tubing T0 with a smaller I.D. of about 50 μm, was used to feed the polyamine at a feeding rate of 0.3 mL/h to balance the production efficiency and the size of the microcapsules. As shown in Figure 2c, the morphology and structure of the prepared microcapsules are almost the same as those prepared by using T1/T2, while their size (85 ± 11 μm) is smaller than that prepared by T1/T2 (Figure 3a). Figure 3b shows the TGA curves of these three microcapsules of different sizes. Since the weight-loss temperatures of the two polyamines in the core, i.e., TEPA and T403, partially overlap that of the polyurea shell of the microcapsule, the precise composition of these three microcapsules cannot be obtained directly from the TGA curves. However, the relative position of the TGA curves can tell the relative composition of these microcapsules. It can be seen that the TGA curves of the two microcapsules prepared using T1/T2 almost overlap each other, while that of the smaller microcapsules prepared using T0/T2 indicates a bigger weight loss at the early-stage (less than 100 °C). It suggests that a higher percentage of the core polyamine in the small-size microcapsules diffuses out and reacts with HMDI to form the polyurea shell, resulting in an increased amount of the low-boiling-point solvents back-flowing into the microcapsules. As a consequence of this, a bigger weight loss appears in the early low-temperature stage of the TGA testing for the smaller microcapsules.

Based on the above results, it can be seen that the microencapsulation technique via integrating microfluidic T-junction and rapid interfacial polymerization is still capable of fabricating small-size microcapsules containing polyamines by changing the T-junction units and varying the feeding rate for the polyamine. According to the studies by Jin et al. [28] and Zhang et al. [19,38,39], the microcapsule size of about 100 μm can balance the manufacturing of self-healing materials and the healing performance of the manufactured self-healing materials. In addition, although this microencapsulation technique is feasible to fabricate microcapsules with a size smaller than 100 μm, it has a relatively low production efficiency for the polyamine microcapsules and the effective core fraction of the fabricated microcapsules is relatively low, the latter, of which is detrimental to the healing performance. Therefore, in the following study of this investigation, the T-junction T1/T2 with feeding rates of 0.3 mL/h and 15.0 mL/h, respectively, for the polyamine and the co-flow solvent was adopted to study the property regulation of the polyamine microcapsules with a size of about 100 μm.

It can be seen from Figure 2 that the outer wall of the prepared microcapsules is thick, rough, and fluffy when the optimized condition for preparing large-size microcapsules was used [36,40]. In addition, the microcapsule collection contains more debris. These are not conducive to the mixing and dispersion of the microcapsules in the resin matrix and affect the self-healing performance of the formulated self-healing materials. Therefore, the following sections were carried out focusing on the property regulation of the small-size polyamine microcapsules.

### 3.2. Influence of Shell-Forming Monomer in Reaction Solution

According to our previous research, [36] the shell-forming monomer (HMDI) in the reaction solution significantly influences the thickness and compactness of the microcapsule shell. While a lower concentration of HMDI causes the microcapsule shell to be too thick and loose, a higher one results in a thinner and too dense shell, which cannot completely cover the secondary microcapsules formed by self-emulsification. The fracture of the directly exposed secondary microcapsules during storage makes the collected microcapsules wet in appearance. However, according to Figure 2d, no evident secondary microcapsules were found when the microcapsules’ size to be prepared is small. In this case, the HMDI concentration in the reaction solution was increased to decrease the thick loose outer wall of the small-size microcapsules and the debris in the microcapsule collection.

**Figure 4:**
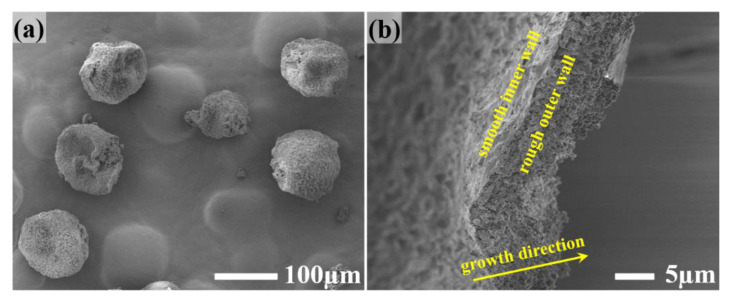
SEM images of the synthesized polyamine microcapsules (**a**) and the corresponding cross-section (**b**) when 9.0 g HMDI was adopted in the reaction solution.

First, the amount of HMDI in the reaction solution was increased from 6.0 g to 9.0 g. It shows that the fabricated microcapsules are still dry and free-flowing, which is completely different from the situation in preparing large-size microcapsules containing pure TEPA. For pure TEPA, when HMDI exceeds 8.0 g, the big microcapsules become wet during storage, attributed to the fracture of the directly exposed secondary microcapsules. Figure 4 shows the appearance (Figure 4a) and the shell cross-section (Figure 4b) of the microcapsules prepared under this condition. It can be seen that by increasing the HMDI content, the fluffy structure on the microcapsules is significantly less than that on the microcapsules prepared with 6.0 g HMDI (Figure 2). Moreover, the outer wall is relatively uniform (Figure 4b). Therefore, it can be considered that increasing the HMDI content in the reaction solution can improve the microcapsule appearance to a certain extent.

The amount of HMDI in the reaction solution was further increased to 12.0 g. The fabricated microcapsules were still dry and have good dispersibility. However, they are much similar to the microcapsules prepared using 9.0 g HMDI regarding their appearance and morphology. Given this, no experiment was conducted to further increase the HMDI concentration to improve the microcapsules’ properties in this study.

### 3.3. Influence of Solvent in Reaction Solution

**Figure 5:**
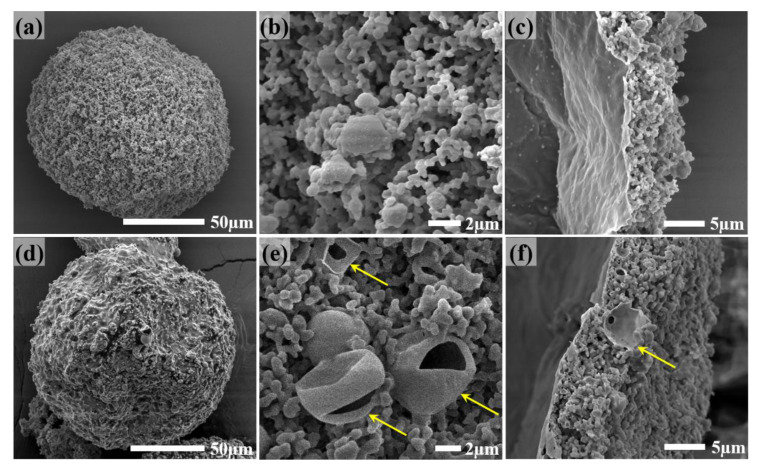
Appearance, surface morphology, and cross-section of the polyamine microcapsules fabricated using solvents with different polarities. (**a**–**c**) 50C10–50C16 mixed solvent, and (**d**–**f**) pure C16. Arrows in (**e**,**f**) indicate the directly exposed secondary microcapsules after fracturing during storage.

**Figure 6:**
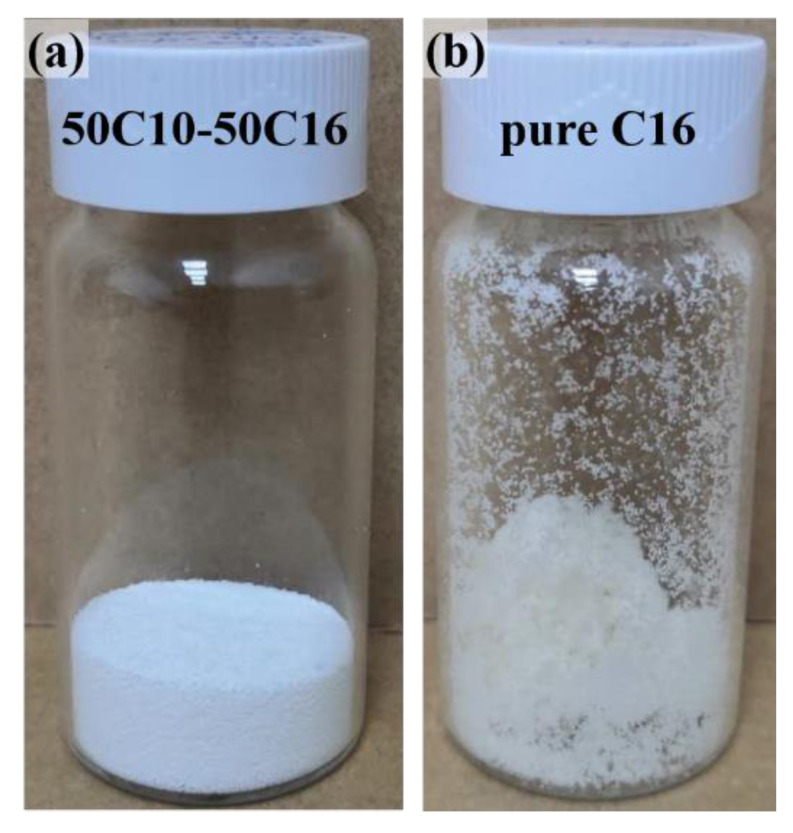
Appearance of the microcapsules fabricated using solvents with different polarities. (**a**) 50C10–50C16 mixed solvent, and (**b**) pure C16.

The polarity of the reaction solution during the formation of the preliminary polyamine microcapsules significantly influences the formed microcapsule shell, thereby affecting the quality of the final microcapsules [36]. Therefore, to further regulate the properties of the small-size polyamine microcapsules, this study chose to use *n*-hexadecane (C16), a nonpolar solvent with polarity lower than C10, to partially or completely replace the solvent C10 in the reaction solution. Dry and free-flowing microcapsules can still be obtained using a mixed solvent with a volume ratio for C10 to C16 of 1:1 (50C10–50C16). Although the turbidity of the reaction solution during the microencapsulation process is still more serious than that during the preparation of large-size microcapsules, it is significantly lighter than the above experiments. Figure 5a–c shows the SEM images of the polyamine microcapsules fabricated by using the mixed solvent. It can be seen that the outer wall of the microcapsule is significantly denser than those of the microcapsules shown in Figure 2 and Figure 4. The fluffy structure is significantly reduced (Figure 5a,b), and the microcapsule shell is thinner (Figure 5c). To further increase the compactness of the outer wall, C10 in the reaction solution was completely substituted by C16 to reduce the polarity of the reaction solution. Similar to the previous study, [36,37,40] the newly collected microcapsules were dry and free-flowing; however, they gradually became wet during their storage. Figure 6 shows the appearance of the microcapsules fabricated by using 50C10–50C16 and pure C16 as the solvent of the reaction solution. The former is dry and can flow freely like sand, while the latter is a little sticky. Nevertheless, they do not aggregate or collapse. Figure 5d–f shows the SEM images of the microcapsule appearance, the surface morphology after washing and drying with deionized water, and the cross-section of the microcapsule shell. It can be seen that there are fractured secondary microcapsules outside the microcapsule (indicated by arrows in Figure 5e,f). Although the wet microcapsules can still be normally used as they retain the majority of the polyamine well in the main chamber and can be scooped out using a spatula, they will adversely affect their performance in fabricating self-healing materials.

Compared to the microencapsulation of pure TEPA, [36,40] the microencapsulation of the mixed polyamine 25TEPA75T403 can be successfully achieved using a reaction solution with significantly reduced polarity. During encapsulating pure TEPA, the microcapsules turn wet when the fed amount of the co-flow solvent (C16 with 1 wt% Arlacel P135) increased to 15.0 mL or 18.0 mL [40]. At these points, the volume ratio of C10 to C16 in the reaction solution is still much bigger than 1:1. In this study, dry and free-flowing microcapsules containing 25TEPA75T403 can even be achieved using the mixed solvent 50C10–50C16 with lower polarity. The reason for this lies in that the polarity of T403 is lower than that of TEPA, and its solubility in C16 is also higher than that of TEPA. Therefore, the polarity of the reaction solution can be reasonably increased. However, the use of pure C16 as the solvent in the reaction solution to further reduce the polarity of the reaction solution still causes the direct exposure of the generated secondary microcapsules in the outer wall of the microcapsules, resulting in the leakage of the core liquid in the secondary microcapsules and the wet appearance.

**Figure 7:**
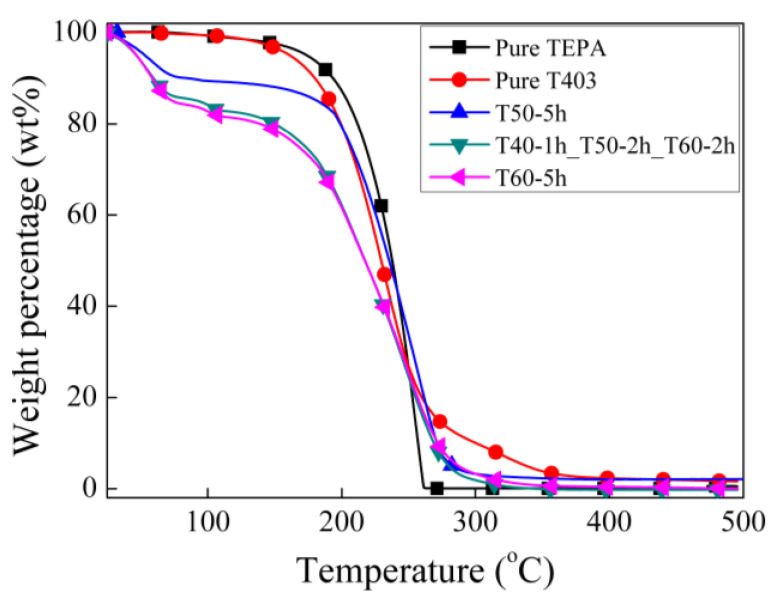
TGA curves of the microcapsules synthesized at different temperatures.

Besides the shell morphology and structure, the TGA test was carried out on the microcapsules prepared with the solvent 50C10–50C16 (the curve indicated by T40-1h_T50-2h_T60-2h in Figure 7). Compared with the microcapsules prepared using pure C10 as the solvent (the curve indicated by 0.3 mL/h for T1/T2 in Figure 3b), the effective core fraction of the microcapsules is only slightly increased. The reason why the core content does not change evident may be that the change of the microcapsule shell is mainly the loose outer part. Although the thickness and porosity of the microcapsule vary to a certain extent, they do not significantly affect the microcapsule composition.

### 3.4. Influence of Reaction Condition

When the microcapsules are large, they need to be synthesized at higher reaction temperatures for a longer duration to obtain a sufficiently thick shell to support the microcapsules. The combination of the reaction temperature and the corresponding duration used in the foregoing experiment is the optimized combination for preparing large-size polyamine microcapsules [40]. In theory, smaller microcapsules only need thinner shells to produce greater strength to support them. Therefore, with the purpose of retaining as much effective core content as possible, the effect of the reaction conditions on the properties of the small-size microcapsules was studied.

**Figure 8:**
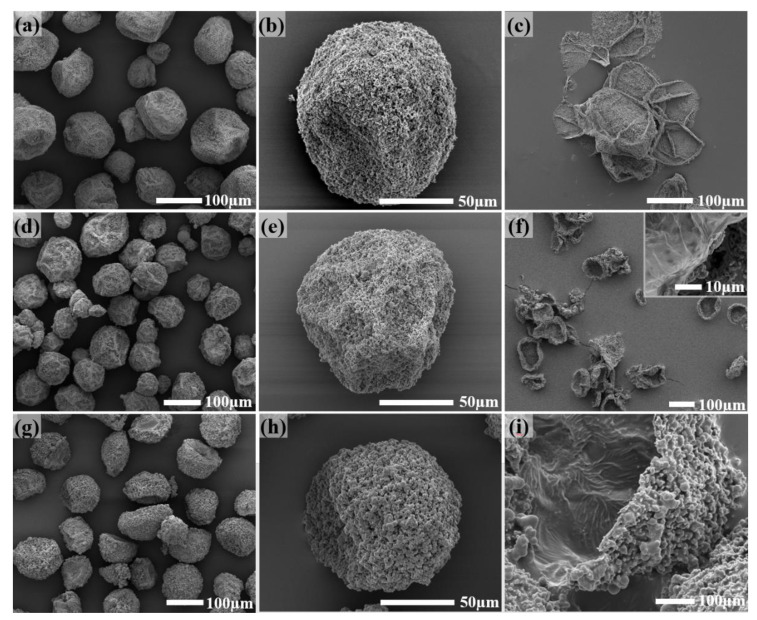
SEM images of the microcapsules were obtained at different reaction conditions. (**a**–**c**) 5 h at 50 °C, (**d**–**f**) 40 °C for 1 h, 50 °C for 2 h, and 60 °C for 2 h in order, and (**g**–**i**) 60 °C for 5 h using T50-5h.

As shown in Table 1, in addition to the reaction condition using stepwise increased temperatures, i.e., 40 °C for 1 h, 50 °C for 2 h, and 60 °C for 2 h in order (T40-1h_T50-2h_T60-2h), other reaction conditions including 50 °C for 5 h (T50-5h) and 60 °C for 5 h (T60-5h) were also adopted in this study. Figure 8a–i, respectively, show the SEM images of the microcapsules prepared under these three conditions. It can be seen that good microcapsules with less coarse structure can be prepared under all three conditions. With the increased reaction duration at higher temperatures, the sphericity of the microcapsules gradually decreased (Figure 8a,d,g), and the coarse and granular polyurea on the surface of the microcapsules gradually increased (Figure 8b,e,h). When the reaction condition is T60-5h, the surface is almost covered by coarse polyurea particles. Figure 8c,f,i respectively show the SEM images of these three microcapsules after being cut with a blade and cleaned. It can be seen that the microcapsules synthesized using T50-5h completely collapse and are not able to maintain the original spherical shape after the removal of the core material (Figure 8c). For the microcapsules synthesized using T40-1h_T50-2h_T60-2h, they can partially maintain their original shape (Figure 8f). Moreover, for these synthesized using T60-5h, they can maintain their original shape, attributed to the strong support of the coarse polyurea particles on the outer shell of the microcapsules (Figure 8i). Consequently, increasing the reaction duration at higher temperatures can increase the shell thickness and, therefore, the mechanical properties of the microcapsules, which is beneficial to their practical application in the storage, use, and service of the microcapsule.

In actual use, it is necessary to retain as much core material as possible because the function of the microcapsule-based functional material depends on the percentage of the functional core material in the microcapsule while maintaining the basic mechanical properties of the microcapsules. In fact, it is difficult to balance them since a higher core fraction and a thicker shell to provide enough strength cannot be achieved at the same time. Figure 7 shows the TGA curves of the microcapsules prepared under these three conditions. Compared with the microcapsules prepared using the stepwise increased temperature (T40-1h_T50-2h_T60-2h) and T60-5h, the microcapsules prepared using T50-5h retains a higher core fraction of the polyamine curing agent. Although the microcapsules prepared using T60-5h have thicker shells than those using T40-1h_T50-2h_T60-2h, the TGA curves are much similar to each other, indicating a similar composition for the two microcapsules. Although the microcapsules prepared using T50-5h have shells not thick enough to support the microcapsule shape alone, they have good tightness and can flow freely. Therefore, it can be considered that it meets the requirements for practical application. In addition, if microcapsules with higher mechanical strength are required in actual use, the microcapsules prepared using T40-1h_T50-2h_T60-2h can be considered.

## 4. Conclusions

This investigation studies the feasibility of using the microencapsulation technique via integrating microfluidic T-junction and interfacial polymerization to prepare small-size microcapsules containing polyamine 25TEPA75T403 and the property regulation of the small-size polyamine microcapsules according to the requirements for practical application. It demonstrates that polyamine microcapsules with sizes around or smaller than 100 μm can be successfully fabricated using the novel microencapsulation technique by using suitable T-junction units and adjusting the feeding rate ratio of the polyamine to the co-flow solvent. Polyamine microcapsules smaller than 100 μm can also be achieved by the technique at the cost of the production efficiency by using a lower feeding rate for the polyamine. The morphology, shell structure, and composition of the small-size microcapsules were regulated by varying the concentration of the shell-forming monomer (HMDI) and the polarity of the reaction solution at the shell-forming state, and the reaction condition at the shell-growth stage. It shows that when encapsulating 25TEPA75T403, the microencapsulation technique can fabricate dry and free-flowing small-size microcapsules using a reaction solution with a higher HMDI concentration and/or a lower polarity, compared to the condition of encapsulating pure TEPA to fabricate large-size microcapsules of about 200 μm. The synthesized microcapsules have relatively denser and thinner shells with less coarse polyurea particles. The mechanical properties of the microcapsules can be adjusted by changing the reaction condition. A longer reaction time at higher temperatures is beneficial to the strength of the microcapsules. In general, the effective core fraction of the polyamine in the microcapsules does not vary much with the composition of the reaction solution and the reaction conditions, though it is relatively higher for the microcapsules fabricated at 50 °C for 5 h. The results of this research will promote the further practical development of self-healing systems based on the microencapsulated epoxy-amine chemistry.

## Figures and Tables

**Table 1:** Experimental parameters adopted in this investigation ^a^.

Table	Feeding Rate for Polyamine (*V*, mL/h)	HMDI (*M*, g)	Solvent ^b^ (*S*)	Reaction Condition ^c^
T1/T2	**0.6**	6.0	C10	T40-1h_T50-2h_T60-2h
T1/T2	**0.3**	6.0	C10	T40-1h_T50-2h_T60-2h
**T0/T2**	0.3	6.0	C10	T40-1h_T50-2h_T60-2h
T1/T2	0.3	**9.0**	C10	T40-1h_T50-2h_T60-2h
T1/T2	0.3	**12.0**	C10	T40-1h_T50-2h_T60-2h
T1/T2	0.3	9.0	**50C10–50C16**	T40-1h_T50-2h_T60-2h
T1/T2	0.3	9.0	**C16**	T40-1h_T50-2h_T60-2h
T1/T2	0.3	9.0	C16-C10	**T50-5h**
T1/T2	0.3	9.0	C16-C10	**T60-5h**

^a^ Other parameters for all the microencapsulation processes, including the co-flow solvent: C16 with 1.0 wt% surfactant Arlacel P135; feeding rate for the co-flow solvent: 15 mL/h; reaction solution: 50.0 mL solvent with 1.0 wt% Arlacel P135, 0.5 wt% catalyst DABCO, and 6.0 g HMDI as the shell-forming monomer; ^b^ C10: decalin; C16: n-hexadecane; ^c^ T40-1h_T50-2h_T60-2h means 40 °C for 1 h, 50 °C for 2 h, and 60 °C for 2 h in order.

## Data Availability

The data presented in this study are available on reasonable request from the corresponding author. The data are not publicly available due to that the data may be further processed for other purposes.

## References

[1] White S.R., Sottos N.R., Geubelle P.H., Moore J.S., Kessler M.R., Sriram S.R., Brown E.N., Viswanathan S. (2001). Autonomic healing of polymer composites. Nature.

[2] Davis D.A., Hamilton A., Yang J.L., Cremar L.D., Van Gough D., Potisek S.L., Ong M.T., Braun P.V., Martinez T.J., White S.R. (2009). Force-induced activation of covalent bonds in mechanoresponsive polymeric materials. Nature.

[3] White S.R., Moore J.S., Sottos N.R., Krull B.P., Santa Cruz W.A., Gergely R.C.R. (2014). Restoration of Large Damage Volumes in Polymers. Science.

[4] Li W., Matthews C.C., Yang K., Odarczenko M.T., White S.R., Sottos N.R. (2016). Damage Detection: Autonomous Indication of Mechanical Damage in Polymeric Coatings. Adv. Mater..

[5] Patrick J.F., Robb M.J., Sottos N.R., Moore J.S., White S.R. (2016). Polymers with autonomous life-cycle control. Nature.

[6] Zhang H., Zhang X., Bao C., Li X., Duan F., Friedrich K., Yang J. (2019). Skin-Inspired, Fully Autonomous Self-Warning and Self-Repairing Polymeric Material under Damaging Events. Chem. Mater..

[7] Cohades A., Branfoot C., Rae S., Bond I., Michaud V. (2018). Progress in Self-Healing Fiber-Reinforced Polymer Composites. Adv. Mater. Interfaces.

[8] Kanu N.J., Gupta E., Vates U.K., Singh G.K. (2019). Self-healing composites: A state-of-the-art review. Compos. Pt. A Appl. Sci. Manuf..

[9] Christopher J.E.P., Sultan M.T.H., Selvan C.P., Irulappasamy S., Mustapha F., Basri A.A., Safri S.N.A. (2020). Manufacturing challenges in self-healing technology for polymer composites—A review. J. Mater. Res. Technol. JMRT.

[10] Zhang H., Zhang X., Bao C., Li X., Sun D., Duan F., Friedrich K., Yang J. (2018). Direct microencapsulation of pure polyamine by integrating microfluidic emulsion and interfacial polymerization for practical self-healing materials. J. Mater. Chem. A.

[11] Wu D.Y., Meure S., Solomon D. (2008). Self-healing polymeric materials: A review of recent developments. Prog. Polym. Sci..

[12] Zhu D.Y., Rong M.Z., Zhang M.Q. (2015). Self-healing polymeric materials based on microencapsulated healing agents: From design to preparation. Prog. Polym. Sci..

[13] Ilyaei S., Sourki R., Akbari Y.H.A. (2020). Capsule-based healing systems in composite materials: A review. Crit. Rev. Solid State Mater. Sci..

[14] Brown E.N., Sottos N.R., White S.R. (2002). Fracture testing of a self-healing polymer composite. Exp. Mech..

[15] Rule J.D., Sottos N.R., White S.R. (2007). Effect of microcapsule size on the performance of self-healing polymers. Polymer.

[16] Zhang H., Yang J. (2014). Development of self-healing polymers via amine–epoxy chemistry: II. Systematic evaluation of self-healing performance. Smart. Mater. Struct..

[17] Kosarli M., Bekas D.G., Tsirka K., Baltzis D., Vaimakis-Tsogkas D.Τ., Orfanidis S., Papavassiliou G., Paipetis A.S. (2019). Microcapsule-based self-healing materials: Healing efficiency and toughness reduction vs. capsule size. Compos. Pt. B Eng..

[18] Yin T., Zhou L., Rong M.Z., Zhang M.Q. (2008). Self-healing woven glass fabric/epoxy composites with the healant consisting of micro-encapsulated epoxy and latent curing agent. Smart. Mater. Struct..

[19] Yuan Y.C., Ye Y., Rong M.Z., Chen H., Wu J., Zhang M.Q., Qin S.X., Yang G.C. (2011). Self-healing of low-velocity impact damage in glass fabric/epoxy composites using an epoxy–mercaptan healing agent. Smart Mater. Struct..

[20] Wei H.G., Wang Y.R., Guo J., Shen N.Z., Jiang D.W., Zhang X., Yan X.R., Zhu J.H., Wang Q., Shao L. (2015). Advanced micro/nanocapsules for self-healing smart anticorrosion coatings. J. Mater. Chem. A.

[21] Shchukin D.G., Möhwald H. (2007). Self-Repairing Coatings Containing Active Nanoreservoirs. Small.

[22] Blaiszik B.J., Sottos N.R., White S.R. (2008). Nanocapsules for self-healing materials. Compos. Sci. Technol..

[23] Samadzadeh M., Boura S.H., Peikari M., Kasiriha S.M., Ashrafi A. (2010). A review on self-healing coatings based on micro/nanocapsules. Prog. Org. Coat..

[24] McIlroy D.A., Blaiszik B.J., Caruso M.M., White S.R., Moore J.S., Sottos N.R. (2010). Microencapsulation of a Reactive Liquid-Phase Amine for Self-Healing Epoxy Composites. Macromolecules.

[25] Jin H.H., Mangun C.L., Stradley D.S., Moore J.S., Sottos N.R., White S.R. (2012). Self-healing thermoset using encapsulated epoxy-amine healing chemistry. Polymer.

[26] Li Q., Siddaramaiah, Kim N.H., Hui D., Lee J.H. (2013). Effects of dual component microcapsules of resin and curing agent on the self-healing efficiency of epoxy. Compos. Pt. B Eng..

[27] Zhang H., Yang J. (2013). Etched glass bubbles as robust micro-containers for self-healing materials. J. Mater. Chem. A.

[28] Jin H.H., Mangun C.L., Griffin A.S., Moore J.S., Sottos N.R., White S.R. (2014). Thermally Stable Autonomic Healing in Epoxy using a Dual-Microcapsule System. Adv. Mater..

[29] Neuser S., Chen P.W., Studart A.R., Michaud V. (2014). Fracture Toughness Healing in Epoxy Containing Both Epoxy and Amine Loaded Capsules. Adv. Eng. Mater..

[30] Zhang H., Wang P., Yang J. (2014). Self-healing epoxy via epoxy–amine chemistry in dual hollow glass bubbles. Compos. Sci. Technol..

[31] Yi H., Deng Y.H., Wang C.Y. (2016). Pickering emulsion-based fabrication of epoxy and amine microcapsules for dual core self-healing coating. Compos. Sci. Technol..

[32] Hu H., Zhang L., Yu R., Yuan L., Yang Y., He X., Wang J., Li Z. (2020). Microencapsulation of ethylenediamine and its application in binary self-healing system using dual-microcapsule. Mater. Des..

[33] Li Q., Mishra A.K., Kim N.H., Kuila T., Lau K.-t., Lee J.H. (2013). Effects of processing conditions of poly(methylmethacrylate) encapsulated liquid curing agent on the properties of self-healing composites. Compos. Pt. B Eng..

[34] Lu X., Katz J.S., Schmitt A.K., Moore J.S. (2018). A Robust Oil-in-Oil Emulsion for the Nonaqueous Encapsulation of Hydrophilic Payloads. J. Am. Chem. Soc..

[35] Yuan L.Y., Sun T.Q., Hu H.L., Yuan S.X., Yang Y., Wang R.G., Lyu C.X., Yang F., Lyu X.X. (2019). Preparation and Characterization of Microencapsulated Ethylenediamine with Epoxy Resin for Self-healing Composites. Sci. Rep..

[36] Zhang H., Zhang X., Chong Y.B., Peng J., Fang X., Yan Z., Liu B., Yang J. (2019). Shell Formation Mechanism for Direct Microencapsulation of Nonequilibrium Pure Polyamine Droplet. J. Phys. Chem. C.

[37] Yang Z., Fang X., Peng J., Cao X., Liao Z., Yan Z., Jiang C., Liu B., Zhang H. (2020). Versatility of the microencapsulation technique via integrating microfluidic T-Junction and interfacial polymerization in encapsulating different polyamines. Colloid. Surf. A.

[38] Yuan Y.C., Ye X.J., Rong M.Z., Zhang M.Q., Yang G.C., Zhao J.Q. (2011). Self-healing epoxy composite with heat-resistant healant. ACS Appl. Mater. Interfaces.

[39] Ye X.J., Zhu Y., Yuan Y.C., Song Y.X., Yang G.C., Rong M.Z., Zhang M.Q. (2017). Improvement of fatigue resistance of epoxy composite with microencapsulated epoxy-SbF5 self-healing system. Express Polym. Lett..

[40] Cao X.W., Peng J.J., Fang X.L., Yang Z.T., Liao Z.C., Yan Z.B., Jiang C.X., Liu B., Zhang H. (2020). Process regulation for encapsulating pure polyamine via integrating microfluidic T-junction and interfacial polymerization. J. Polym. Sci..

